# Lipo-Chitin Oligosaccharides, Plant Symbiosis Signalling Molecules That Modulate Mammalian Angiogenesis *In Vitro*

**DOI:** 10.1371/journal.pone.0112635

**Published:** 2014-12-23

**Authors:** Michael A. Djordjevic, Anna Bezos, Susanti, Laurence Marmuse, Hugues Driguez, Eric Samain, Boris Vauzeilles, Jean-Marie Beau, Farzaneh Kordbacheh, Barry G. Rolfe, Ralf Schwörer, Alison M. Daines, Peter M. Gresshoff, Christopher R. Parish

**Affiliations:** 1 Research School of Biology, Plant Science Division, College of Medicine, Biology and the Environment, Australian National University, Canberra, ACT, Australia; 2 John Curtin School of Medical Research, College of Medicine, Biology and the Environment, Australian National University, Canberra, ACT, Australia; 3 University Grenoble Alpes, CERMAV, Grenoble, France CNRS, CERMAV, Grenoble, France; 4 University Paris Sud, Institut de Chimie Moléculaire et des Matériaux d’Orsay, Orsay, France, and Centre de Recherche de Gif, Institut de Chimie des Substances Naturelles du CNRS, Gif-sur-Yvette, France; 5 Ferrier Research Institute, Victoria University of Wellington, Lower Hutt Wellington, New Zealand; 6 The Centre for Integrative Legume Research, The University of Queensland, St Lucia, Brisbane, Queensland, Australia; Medical College of Wisconsin, United States of America

## Abstract

Lipochitin oligosaccharides (LCOs) are signaling molecules required by ecologically and agronomically important bacteria and fungi to establish symbioses with diverse land plants. In plants, oligo-chitins and LCOs can differentially interact with different lysin motif (LysM) receptors and affect innate immunity responses or symbiosis-related pathways. In animals, oligo-chitins also induce innate immunity and other physiological responses but LCO recognition has not been demonstrated. Here LCO and LCO-like compounds are shown to be biologically active in mammals in a structure dependent way through the modulation of angiogenesis, a tightly-regulated process involving the induction and growth of new blood vessels from existing vessels. The testing of 24 LCO, LCO-like or oligo-chitin compounds resulted in structure-dependent effects on angiogenesis *in vitro* leading to promotion, or inhibition or nil effects. Like plants, the mammalian LCO biological activity depended upon the presence and type of terminal substitutions. Un-substituted oligo-chitins of similar chain lengths were unable to modulate angiogenesis indicating that mammalian cells, like plant cells, can distinguish between LCOs and un-substituted oligo-chitins. The cellular mode-of-action of the biologically active LCOs in mammals was determined. The stimulation or inhibition of endothelial cell adhesion to vitronectin or fibronectin correlated with their pro- or anti-angiogenic activity. Importantly, novel and more easily synthesised LCO-like disaccharide molecules were also biologically active and de-acetylated chitobiose was shown to be the primary structural basis of recognition. Given this, simpler chitin disaccharides derivatives based on the structure of biologically active LCOs were synthesised and purified and these showed biological activity in mammalian cells. Since important chronic disease states are linked to either insufficient or excessive angiogenesis, LCO and LCO-like molecules may have the potential to be a new, carbohydrate-based class of therapeutics for modulating angiogenesis.

## Introduction

Lipochitin oligosaccharides (LCOs) are natural products synthesised by prokaryotic nitrogen fixing bacteria (generically called “rhizobia”) and eukaryotic arbuscular mycorrhizal (AM) fungi. These naturally occurring molecules act as microbial signals that are required to establish nitrogen-fixing nodule formation and mycorrhization respectively. These symbiotic interactions are of paramount ecological and agronomic importance and contribute a significant proportion of biologically-available nitrogen and phosphorous to agronomic systems respectively [1], [2]. The legume-rhizobial symbiosis evolved relatively recently (*circa* 60 million years ago) whereas the AM symbiosis is more ancient (*circa* 460 million years old) and widespread and there is considerable genetic overlap in the signal transduction pathways that underpin both interactions [3]. Since 70–90% of ancient and recently-evolved land plants can establish mycorrhization, LCO recognition by plants is widespread [1], [3].

Rhizobial and fungal LCOs comprise of three to five β-1,4-linked *N*-acetylglucosamine residues. In addition, they have an *N-*linked acyl substitution at the non-reducing end and can often have modifications to the C6 position of the reducing end N-acetly glucosamine residue. Rhizobia synthesise LCOs by first making a de-acetylated chitin-oligomer by the combined action of an oligo-chitin synthase (NODC) and an LCO de-acetylase (NODB), which removes an acetyl group from the non-reducing end residue [4]. Subsequently, the terminal residues can be decorated with assorted substitutions which results in a wide structural diversity of rhizobial LCOs [1], [5] with molecular masses between 1,000–1,500 Da [5]. In legumes, the lengths of the chitin backbone and terminal substitutions greatly influence the extent and specificity of LCO recognition by lysin motif (LysM) type receptors [1], [6], [7], [8]. The LCO substitutions that influence the extent and specificity of recognition leading to symbiotic interactions include non-reducing-end, *N*-linked fatty acids (C16–C18) and reducing-end, *O*-linked sugars or sulphate [1], [5], [6], [7], [9]. Recently, the chitin backbone of the LCO has been shown to interact with its corresponding LysM receptor [7]. In contrast to rhizobial and AM LCOs, different plant LysM-type receptors (*e.g.*, LYK3 and LYK4) preferentially recognise chitin oligomers as microbe-associated molecular patterns (MAMPs) and this induces an innate, ‘general-immune’ response [10], [11], [12]. Moreover, it has been shown that LYK3 in Arabidopsis retains a residual ability to recognise LCOs but instead of innate immunity being induced this interaction results in a suppression of innate immunity [13]. Therefore, although LCOs and chitin oligomers may share an affinity for chitin-motif recognising receptors the down-stream responses induced are different.

Although LCO and, in particular, chitin-like molecules are widespread in nature, mammals are not known to make chitins or chitin-like molecules although they encode chitin synthase genes (*e.g.*, DG42 [14]) which have functions akin to NODC. In addition, like plants, mammals also recognise chitin oligomers as MAMPs, and this also leads to the induction of an innate immune [12], [15], [16] and other responses [17], [18]. Recently, it has been recognised that LysM domain-containing proteins [19] and several glycoside hydrolase (GH) family proteins (*e.g.*, GH18) [20], [21] occur in the genomes of vertebrates but their functions have not been fully elucidated. The ligands for the mammalian LysM-domain containing proteins are not known but the GH family proteins have chitin-binding and/or chitinase activity. One GH protein, YKL-40, is a growth factor that induces cell proliferation by activating protein kinase signalling and YKL-40 over-expression enhances the migration of human macrophage and endothelial cells [22], [23]. In contrast to mammals, cyprinid fish not only appear to synthesise chitin oligosaccharides *de novo*, but chitooligosacharides also appear to play a role in their early development [14], [24]. Chitin recognition leads to induction of innate immunity and there are reports that medium sized chitin macro-particles (but not small-sized particles) may be linked to a possible role for these chitin particles in asthma and allergy pathogenesis [25]. However, to our knowledge, no reports have been made of toxicity for short chain chitin-based molecules in mammals or vertebrates and chitins and chitosans are commonly used as dietary supplements.

Angiogenesis is a complex, multi-stepped and highly regulated process in mammals that involves endothelial cells (ECs) and has essential roles in development, reproduction and repair. In addition, a plethora of important diseases are associated with excessive or insufficient angiogenesis including solid tumour formation, age-related macular degeneration, diabetic retinopathy, atherosclerosis, peptic ulcers, rheumatoid arthritis, hypertension and ischemic conditions [26], [27], [28], [29]. Therefore, biotechnological research has targeted angiogenesis to identify new therapeutics. Most studies have focused on discovering anti-angiogenic molecules and there has been progress in utilising anti-angiogenic therapies in treating cancer and age related macular degeneration [26]. Several clinically approved anti-angiogenic therapies include the use the anti-angiogenic VEGF antibody (Avastin), VEGF receptor kinase inhibitors (Sunitinib, Pazopanib, Sorafenib and Vandetanib) and the age-related macular degeneration therapeutics ranibizumab and pegaptanib [30]. Pro-angiogenic therapies have received less attention however, there is a pressing need to develop a pipeline of potential pro-angiogenic therapeutics to treat conditions such as coronary and peripheral artery disease especially since clinical trials done so far with existing drug candidates have lacked efficacy [31], [32].

During *in vivo* angiogenesis, the ECs that form new blood vessels from existing vessels re-activate, migrate, proliferate and reorganise into tubes. Several biological assays have been developed to assess candidate angiogenesis therapeutics [33] but not all of them incorporate all of the four EC-associated activities required for forming new blood vessels. However, the rat aorta ring assay, which is widely used as a screen for anti-angiogenic activity, involves EC re-activation, migration, proliferation and reorganisation and as a result of these processes the formation of EC-derived micro-capillaries occur over a longer time course than other biological assays [34]. Therefore, although the rat aorta ring assay is *ex vivo* it is considered to more closely mimic *in vivo* conditions and it has been used to successfully identify the anti-angiogenic therapeutic, Muparfostat (PI-88), which is already in phase III cancer trials [34]. We show here that adjustments to the concentration of heat inactivated foetal bovine serum can be made in this widely used bioassay so that the anti- or pro-angiogenic effects of an added compound on EC cells can be monitored (measured by determining the speed and extent of angiogenesis over time). Other commonly used *in vitro* assays examine the individual component steps of angiogenesis and are used to define the cellular mode-of-action of a particular compound over a shorter time frame [33].

Since plants and animals share the capacity to recognise chitin oligomers as MAMPs [12] and LCOs are recognised by oligo-chitin receptors in Arabidopsis [13], we hypothesized that mammalian cells would also detect LCOs and, if so, that this might reflect a more widespread ability of oligochitin receptors to interact with LCOs in nature. The *ex vivo* rat aorta ring angiogenesis assay was used to assess this possibility. Using this assay, we discovered that certain naturally-occurring LCOs, but not chitin oligomers, affected angiogenesis in a structure-dependent manner *in vitro*. Therefore, we synthesised or assessed a broader range of naturally occurring LCOs or ‘LCO-like’-molecules for angiogenesis-regulating activity to determine the structural requirement for this activity. Structure-activity assays identified the molecular constraints for LCO- or LCO-like molecules to have effects on angiogenesis. The cellular mode-of-action was determined using *in vitro* biological assays that tested individual cellular components of angiogenesis (*e.g.*, EC-reactivation, EC-adhesion, or EC-migration). Finally, since a de-acetylated chitobiose (but not chitobiose) was found to be biologically active, a synthesis was devised to generate novel derivatives of this disaccharide that have LCO-like substitutions as a route to develop a pipeline of potential therapeutics. The results showed that these disaccharides may provide cheaper and more-readily-synthesised candidates for future angiogenesis therapeutics based on the structures of LCOs.

## Materials and Methods

### Animal Ethics

The experiments conducted in this manuscript were performed under the Australian National University Animal Experimentation Ethics Committee stipulation A2011/35. The Institutional ethics committee specifically approved this study.

### Rat aorta ring assays

**Figure 1:**
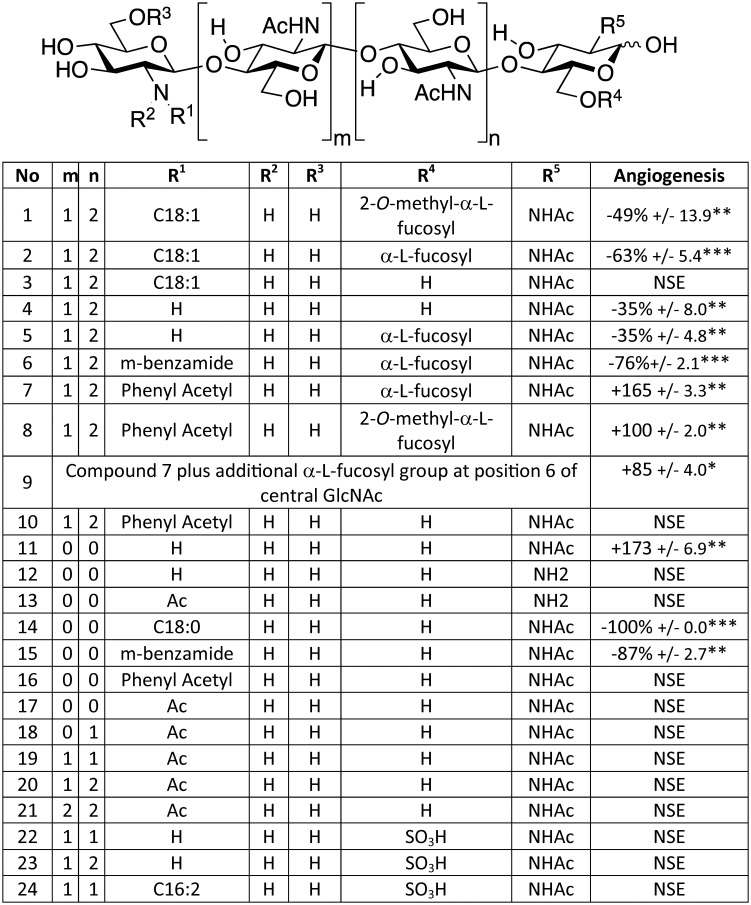
Angiogenesis modulating activity of compounds determined by the rat aorta ring angiogenesis assay. The generic structure indicates the positions of the substitutions, R^1^–R^5^, for the 24 compounds tested. Compounds activities were measured as a % of untreated control: positive values represent enhanced angiogenesis; negative values represent inhibited angiogenesis. PI-88 (Muparfostat; 100 µg/ml) was included in all experiments as an anti-angiogenesis control. This compound is in phase III cancer trials and used therapeutically at these concentrations [34]. In multiple assays PI-88 typically inhibited angiogenesis by 45–65%. The Table includes results for the 24 LCO and LCO-like compounds only which were accumulated from the results of multiple experiments each with mock- and Muparfostat-treated controls. Chitin oligomers were purchased from Sapphire Chemicals and Compound 12 from Ciba Geigy. Compounds 11, 13–16 were synthesised to purity and confirmed by high resolution mass spectrometry and NMR (Fig. S2 and Methods S1 in S1 File). All other compounds were synthesised as described [40], [58]. Compound 11 was also derived independently from the supernatant of transgenic *E. coli*. The benzamide of compound 6 is described in Fig. S1 in S1 File; the benzamide of compound 15 has an *O-*linked aliphatic C_13_H_27_. All compounds were tested at 100 µg/ml unless otherwise stated. NSE = no significant effect. *p<0.05; **p<0.01, ***p<0.001. Compound 24 was provided by Prof. William Broughton (University Genéve, Switzerland).

Aortic segments (1 mm; 9 month-old Fischer rats) were cultured in a gel-fibrin matrix using six replicates per treatment [35]. Vessel outgrowths were microscopically quantified after 5–7 days in the presence/absence of compounds (Fig. 1) in Medium 199 containing 20% or 5% heat inactivated foetal bovine serum (HIFBS; Sigma) for anti-angiogenic or pro-angiogenic activity, respectively [36]. In 20% HIFBS, endothelial cell tubes ramify over 5 days and anti-angiogenic activity retards tube formation prior to day 5. In sub-optimal HIFBS (5%), endothelial cell tubes form over 7 days and pro-angiogenic or anti-angiogenic activity can be observed at days 5, 6 and 7. The compounds tested were dissolved in 50% HPLC grade acetonitrile and diluted at least 1∶100 in supplemented medium M199. Control cultures received medium with the diluent but without the test substance or with the anti-angiogenic control, Muparfostat (PI-88 [34]), at 100 µg/ml, which is its therapeutic dose in anti-cancer trials. The use of Muparfostat in assays represents an additional control. Select compounds were also tested at 10 or 1 µg/ml.

### *In vitro* integrin-mediated attachment and cell migration assays

Human microvessel endothelial cells (HMECs) and human umbilical vein endothelial cells (HUVECs) were assessed for enhanced or inhibited attachment to fibronectin or vitronectin using the Rose Bengal assay [37]. A scratch-wound migration assay was used to test the speed of closure of disrupted confluent HUVEC monolayers [38]. HUVEC (4×10^4^ cells/well) were added to 48-well plates in 300 µl/well of M199 medium, supplemented with 20% HIFBS, 0.24 µg/ml gentamycin, 2 mM L-glutamine, 0.04 mg/ml ECGS (endothelial cell growth supplement) and 0.135 mg/ml heparin and incubated at 37°C in 5% CO_2_ until confluent. The HUVEC monolayers were wounded, rinsed twice with 300 µl/well of culture medium before 300 µl/well of medium was added with, or without, the test compound. Wound recovery was photographed several times daily using an Olympus IX81 CellR microscope and quantified using NIH Image J software. Compounds were tested at 25 µg/ml in these *in vitro* assays.

### LCO and LCO-like molecules

LCO and LCO-like compounds were generated in transgenic *E. coli* [39], [40], [41], [42], [43], [44], [45], [46], [47] unless stated otherwise in Fig. 1 or synthesised as stated below. The structure and purity of the molecules used in this study was confirmed by mass spectrometry as previously described [39], [40], [41], [42], [43], [44], [45], [46], [47]. The purity and integrity of newly described molecules are detailed below.

### Synthesis of compounds 11, 13, 14, 15 and 16

Five disaccharides (compounds 11, 13–16) [48] were synthesised starting from commercially available product **29** (azido sugar 4-methoxyphenyl 2-azido-4,6-*O*-benzylidene-2-deoxy-β-d-glucopyranoside) (Fig. S2 in S1 File). Selective deprotection/*N*-acetylation of product **33** yielded disaccharides β-1-4 GlcNAc-GlcN and β-1-4 GlcN-GlcNAc (compounds 11 and 13; [48] Fig. 1). Compounds 14, 15 and 16 (Fig. 1) were derived from compound 11. Final product structures were verified by ^1^H- and ^13^C-NMR and high resolution mass spectroscopy. Fig. S2 and Methods S1 in S1 File show details of the experimental procedures and analytical data for the synthesis of compounds 11, 13–16.

### Statistical Analysis

Data and results are reported as means ± SE. Statistical significance was measured using two tailed t-test for Fig. 1, or one or two-way ANOVA, as appropriate, using the GraphPad prism 5.04 and Instat software programs (GraphPad Software, San Diego, CA). *P* values less than 0.05 were considered statistically significant.

## Results and Discussion

### Soybean LCOs affect the development of mammalian endothelial cells (ECs) *ex vivo*

Twenty-four LCO, LCO-like or chitin oligomer molecules were synthesised or obtained and quantitatively and qualitatively screened *in vitro* for pro- or anti-angiogenic effects on microvessel growth from rat aorta segments (Fig. 1). The compounds used were fully synthesised (compounds 11, 13–16), or partially synthesised after first purifying precursors from the supernatants of *Escherichia coli* harbouring rhizobial LCO genes (*i.e.* for compounds 1–3, 5–10, 22, 23)[40], [41], isolated directly from the supernatants of *Escherichia coli* harbouring rhizobial LCO genes (compounds 4, 11) [40], [41], obtained from commercial sources (compounds 12, 17–21) or isolated directly from rhizobial secretions (compound 24; Fig. 1; Methods). The LCOs used included naturally-occurring structures with known and specific biological activity for soybean (compounds 1 and 2) or *Medicago* (compound 24). The artificial LCO-like molecules used included de-acetylated chitin pentasaccharide derivatives each with non-naturally occurring m-benzamide- or phenyl-acetyl-substitutions to the non-reducing terminus [40] (Fig. 1; Fig. S1 in S1 File). Using the results from the structure activity relationships of larger LCO molecules, smaller and more-readily-synthesised chito-disaccharides (compounds 11, 13–16) were synthesised and tested (Fig. 1; Methods, Fig. S2 in S1 File).

The well-characterised rat aorta biological assay which generates EC-derived “tubes” akin to micro-capillaries was used to assess the activity of various compounds (Fig. 1). Compounds 1 and 2 were chitopentamers similar to naturally-occurring LCOs that have biological activity on soybean (*e.g.,* compound 1–2) [49] and contained an *N*-linked non-reducing end acylation and a reducing end fucose or methyl-fucose. Compounds 1 and 2 were shown to possess anti-angiogenic activity akin to the control anti-angiogenic and cancer therapeutic compound Muparfostat (also known as PI-88; Fig. 1). Removing the fucosyl substitutions from compounds 1 and 2 rendered the resulting product (compound 3) inactive. In addition, LCO-like compounds comparable to compounds 1 and 2, but which lacked acylation, gave weak anti-angiogenic (compounds 4–5), or nil activity (compounds 23–23). Compounds with sulfate modifications to the reducing terminus, including the *Medicago* active LCO compound 24, were also inactive. Importantly, un-substituted chitin oligomers of two to six residues in length were inactive (compounds 17–21; Fig. 1). The length of the oligo-chitin chain in the un-substituted chitin ologomers mirrored the length of N-acetyl glucosamine residues occurring in the LCO molecules tested. Collectively, these results show that, like plants, mammalian ECs can distinguish between LCOs and chitin oligosaccharides, even though these molecules share an oligo-chitin-like backbone. The results suggest that LCO structural specificity (e.g., acylation and fucosylation) is required for strong anti-angiogenic activity on ECs and the most biologically active LCO molecules strongly resembled structures which affect soybean plants.

### ECs also recognise LCO-like molecules with synthetic acyl groups

**Figure 2:**
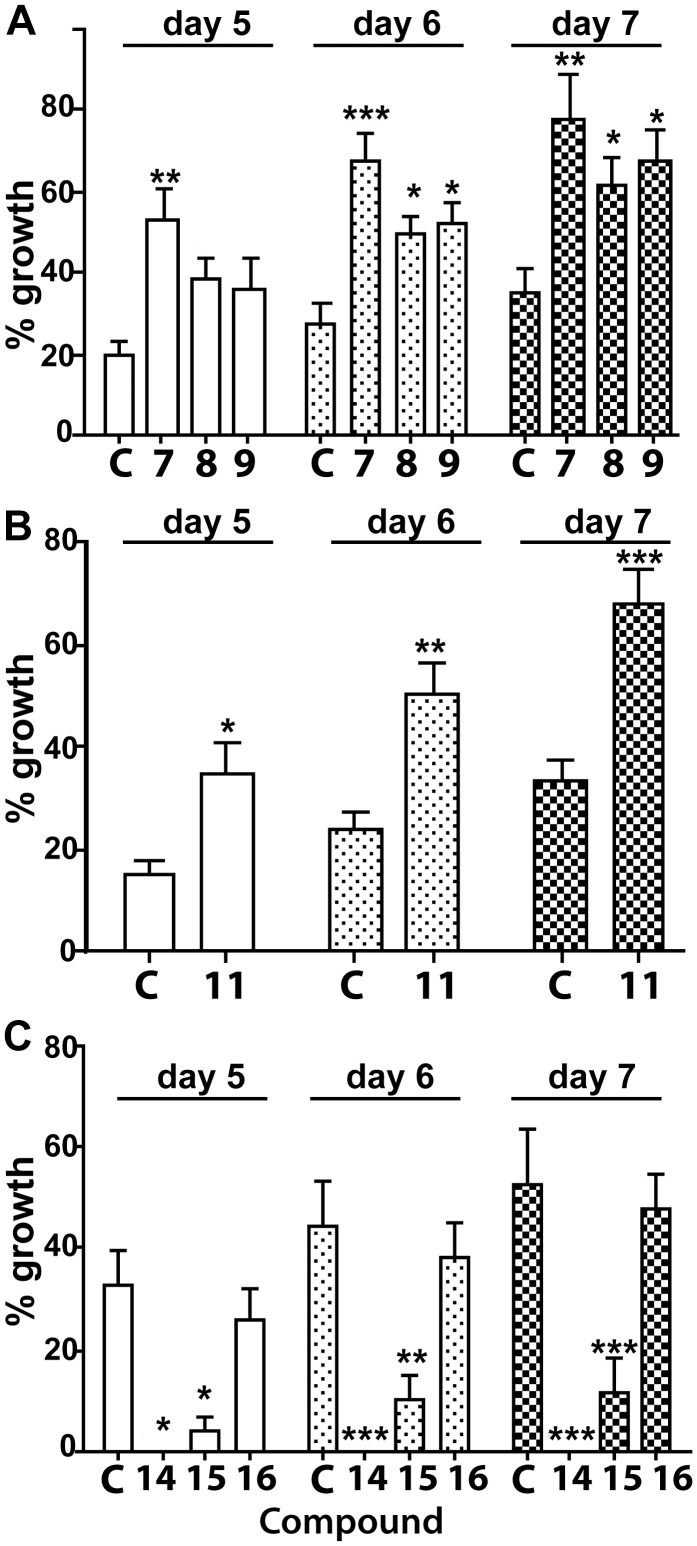
Assessment of the sprouting of tubes from rat aorta rings induced by pro-and anti-angiogenic LCO and LCO-like compounds. (A B) Tube sprouting induced by pro-angiogenic compounds 7–9 (A) compound 11 (B) compared to mock-treated controls (“C”). (C). Tube sprouting induced by the anti-angiogenic disaccharide compounds, 14 and 15, relative to a mock-treated control (“C”) and compound 16. (* = p<0.05; ** = p<0.01; *** = p<0.001; one-way ANOVA).

Since the most potent LCOs possessed a fatty acid substitution to the non-reducing end, we tested if this modification could be substituted by non-naturally-occurring benzamide or phenylacetyl groups as the ability of these molecules bearing these modifications to bind LCO receptors in plants has been demonstrated [40]. These synthetic LCOs were made using transgenic *E. coli* harbouring the rhizobial genes, *nodC* and *nodB*, with and without *nodZ,* to produce the precursor molecules before the artificial groups were added by a separate synthetic step [40]. Strains containing *nodC* and *nodB* do not produce rhizobial-type LCOs but instead produced oligo-chitin-like derivatives (mostly pentamers) devoid of the *N-*acetyl group on the non-reducing terminal residue. NODZ, which is encoded by a transferase gene [49], can add fucose or methylfucose to the reducing end residue but also, occasionally, to an internal *N*-acetylglucosamine (*e.g.*, compound 9; Fig. 1). Purification of these fucosylated or methyfucosylated oligo-chitin-like derivatives enabled the subsequent chemical additions of either m-benzamide or phenylacetyl substitutions to the non-reducing end to generate the synthetic LCO compounds 6–10 (Fig. 1) [40]. Compound 6 (m-benzamide; a mimic of a C16∶2 substitution; Fig. S1 in S1 File) was amongst the most potent anti-angiogenic compounds and its structure and activity was consistently stronger than compounds 1 and 2 and Muparfostat (Fig. 1). By contrast, compounds 7–9, which contain phenylacetyl substitutions instead of a fatty acid or m-benzamide group (Fig. 1), were pro-angiogenic (Fig. 2A; Fig. S1 in S1 File). This indicated that the size and nature of the non-reducing-end substitutions were important for the type of LCO biological activity imparted when these compounds were exposed to mammalian cells. More importantly, by changing the nature of the non-reducing end substitution to a phenylacetyl group instead of a longer acyl derivative, molecules with the opposite biological activity could be generated.

We tested the requirement for reducing end fucosylation for the biological activity of these synthetic LCO-like compounds by generating compound 10 which lacked fucosylation. This compound was inactive (Fig. 1; Fig. S1 in S1 File) and this was consistent with the inactivity of compound 3, which also lacked fucosylation. Therefore, like plants [5], [7], [9], [50], the most biologically active LCO or LCO-like molecules in mammals, required appropriate terminal substitutions and the nature of the substitutions influenced whether anti-angiogenic, pro-angiogenic or inactive molecules were generated.

### A de-acetylated chitobiose is biologically active

Although pentamers of LCO precursors can be readily synthesised by, and extracted from, transgenic *E. coli* [39], they are challenging and expensive to synthesise chemically as they require complex protection and de-protection reactions for each sugar molecule added. In addition, *E. coli* is an unsuitable system for generating potential therapeutics due to the presence of its lipopolysaccharide, which is biologically active in humans [51], and this may contaminate the supernatants of *E. coli* from which LCOs are initially extracted. Therefore, from a synthesis perspective, smaller and simpler LCO-like molecules are desirable. To examine this possibility, compound 11, a de-acetylated disaccharide, was synthesised firstly *in vivo* using *E. coli* harbouring *nodC* and a chitinase [39]. Surprisingly, compound 11 (Gln-GlnAc; β 1–4 linkage), was pro-angiogenic (Fig. 2B, Fig. 1) even though it lacked acyl or sugar substitutions to the termini.

To validate the result obtained with the *E. coli*-derived compound 11, and to examine the structural requirements for its pro-angiogenic activity, compound 11 and its isomer (compound 13; GlnAc-Gln) were chemically synthesised (see Fig. S2 and Methods S1 in S1 File for a detailed description of the synthesis) and compared their activity with that of two other structurally similar disaccharides (compounds 12 and 17). The independently-derived and synthetic compound 11 was as pro-angiogenic as compound 11 isolated from *E. coli* culture secretions. This result not only validated the original result with the *E. coli*-supernatant-derived compound 11 but also negated any possibility of trace *E. coli* molecules, present in the supernatant, such as lipopolysaccharide [51], being involved in the biological activity of the original compound 11. Therefore, these results demonstrate that compound 11, irrespective of whether it was derived from *E. coli* or via chemical synthesis (Fig. 2B; Fig. 1), represents a biologically-active and structurally simple compound. Importantly, this compound is more-readily-synthesisable *in vitro* than the larger LCO molecules made using transgenic *E. coli* derivatives combined with an additional chemical synthesis step.

To determine how specific the activity of compound 11 was, its biological activity was compared to chito-like disaccharides with structural similarity. The activity of compound 11 was specific since three structurally-related isomers were inactive. These included the synthesised compound 13 (GlnAc-Gln, Fig. S2 in S1 File) as well as compound 17 (GlnAc-GlnAc; chitobiose) and compound 12 (Gln-Gln); both compound 17 and 12 are commercially available. The differential activity of compounds 11 (Gln-GlnAc) compared to compounds 12 (Gln-Gln), 13 (GlnAc-Gln) and 17 (GlnAc-GlnAc) demonstrated a specific structural requirement for pro-angiogenic activity. The activity of compound 11 could in part be explained by a reported inability of chitinases to hydrolyse chitobiose [52] and this is consistent with this compound 11 being the dominant product of the *E. coli* strain harbouring *nodC* and a chitinase [39]. In addition, the results clearly demonstrate that de-acetylation of the non-reducing end of compound 11 was a strict requirement for its biological activity and that the de-acetylated chitin backbone was a primary structural basis of recognition.

### Acylated derivatives of compound 11 are biologically active

The activity and structure of compound 11 provided an opportunity to chemically synthesise several non-naturally-occurring, disaccharide structural variants (compounds 14–16; Fig. S2 and Methods S1 in S1 File) based on the LCO and LCO-like molecules we tested with biological activity (*e.g.*, compounds 1 and 2 and 6–10). Compounds 14–16 contained synthetic lipophilic side chains designed to impart a longer retention time *in vivo* than that expected for compound 11. As expected, compounds 14 and 15 were strongly anti-angiogenic (Fig. 2C; Fig. 1) whereas compound 16 was, unexpectedly, inactive (Fig. 2C; Fig. 1). Nevertheless, since compounds 14 and 15 were anti-angiogenic and compound 11 was pro-angiogenic, it is clear that the biological activity of these shorter and novel disaccharides is also affected by the structure and type of substitution occurring at the non-reducing end. The finding of biological activity in mammals with these smaller, easier-to-synthesise disaccharides opens the possibility of producing a range of smaller LCO-like derivatives as potential therapeutics.

### LCOs affect EC cell adhesion to ECM components

**Figure 3:**
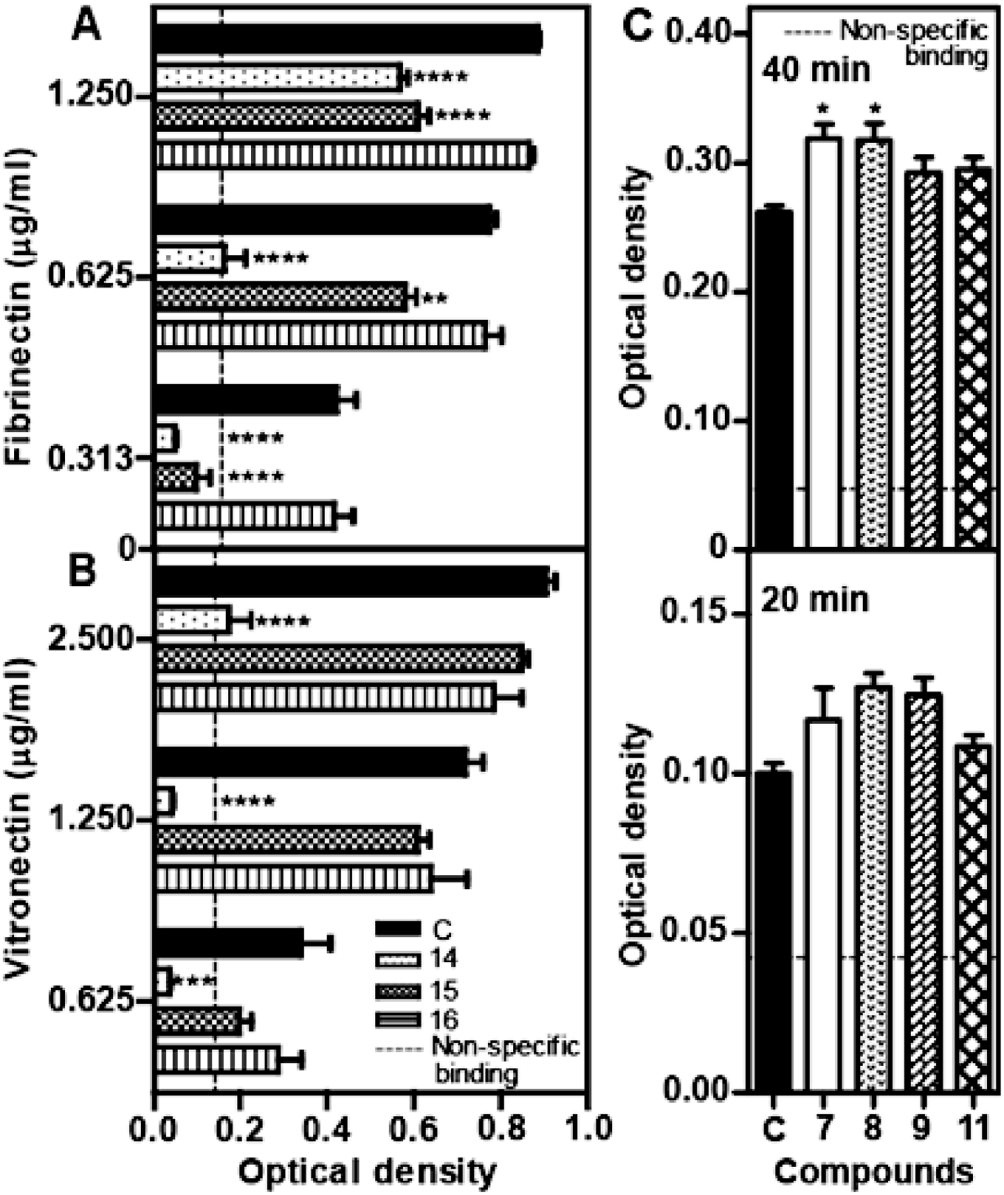
LCO enhancement or inhibition of integrin-mediated attachment of endothelial cells to extracellular matrix components *in vitro.* (A–B) A 2-way ANOVA analyses showed the anti-angiogenic compound 14 inhibits HMEC (human microvascular endothelial cell) attachment to immobilised fibronectin and vitronectin whereas compound 15 affects HMEC attachment to fibronectin only (compounds added at 25 µg/ml, adhesion 60 min). The vitronectin and fibronectin concentrations refer to the concentrations used to coat the plates. (C) One-way ANOVA analyses showed pro-angiogenic compounds 7 and 8 enhances HMEC attachment to vitronectin after incubation for 40 min. “C” designates control in A–C. *(p<0.05); **(p<0.01), ***(p<0.001), ****(p<0.0001). Vertical bars represent SEM (n = 6).

**Figure 4:**
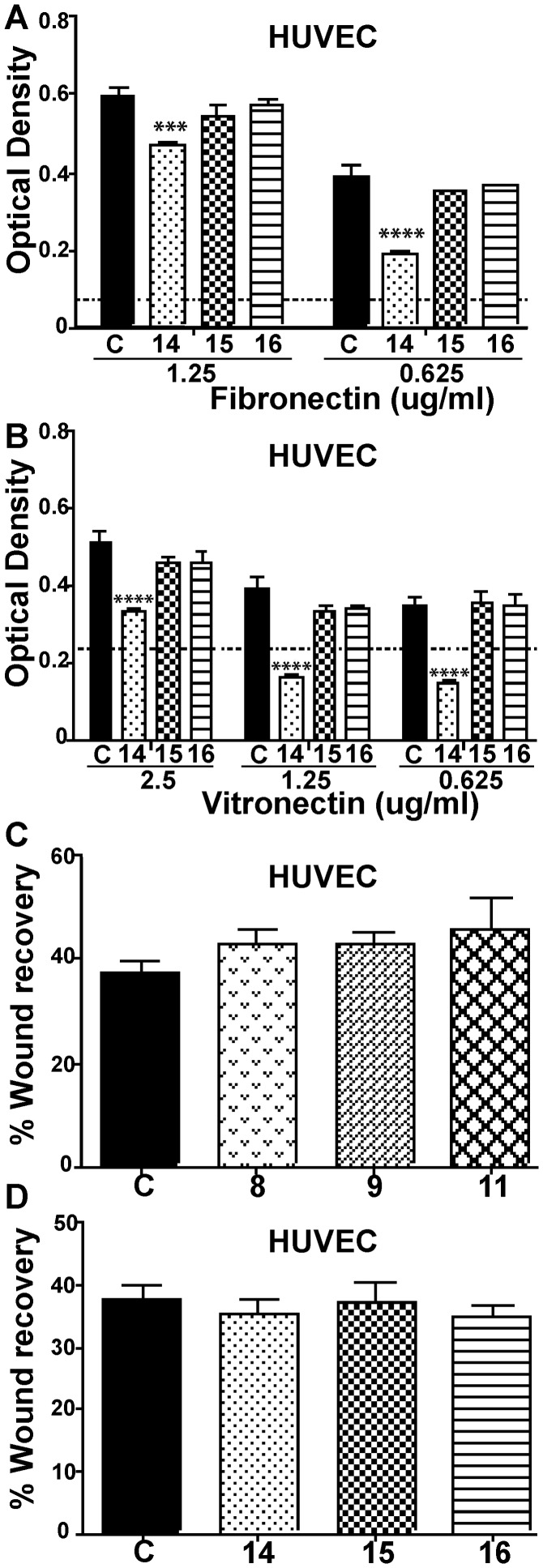
Effects of selected LCO and LCO-like compounds HUVEC adhesion to ECM components and cell migration. (A, B) Effect of compounds 14, 15 and 16 on the attachment of HUVEC to fibronectin-or vitronectin-coated plates, respectively. A 2-way ANOVA analyses showed only compound 14 significantly affected integrin-mediated attachment. (C, D) No compounds tested significantly affected cell migration in a wound recovery assay after disrupted HUVEC monolayers were treated with compounds 8, 9 or 11 or 14, 15 or 16, respectively. “C” designates control in A–D. (*** = p<0.001, **** = p<0.0001; one way ANOVA).

The cellular mode-of-action of selected LCOs was explored using biological assays that emulate individual mechanistic components of angiogenesis [26], [29]. These LCOs were tested *in vitro* for effects on EC adhesion to extracellular matrix (ECM) components (Fig. 3A–C; Fig. 4A–B), a process that is usually integrin dependent [53], and EC migration (Fig. 4A, B). The results suggested that the LCO mode-of-action was mediated through inhibition or enhancement of EC attachment to the ECM components, fibronectin and vitronectin (Fig. 3A–C). For example, the anti-angiogenic compound 14 inhibited adhesion of HMEC (Fig. 3A, B) and HUVEC (Fig. 4A, B) to fibronectin and vitronectin whereas compound 15 inhibited adhesion of HMEC to fibronectin. In contrast, the pro-angiogenic compounds 7–9 and 11, enhanced the rate of HMEC adhesion to vitronectin (Fig. 3C). Therefore, LCO enhancement or inhibition of angiogenesis correlated with their activity in the cell adhesion assay. No compounds tested significantly affected the speed of cell migration in a wound recovery assay *in vitro* after disrupted HUVEC monolayers were treated with several compounds (Fig. 4C–D). Since migration of ECs is one of the earliest steps in angiogenesis, this suggests that enhancement or inhibition of angiogenesis by compounds with biological activity occurred after this step. Of note, compound 14 exhibited a stronger anti-angiogenic activity (Fig. 2C; Fig. 1) than compounds 15 and 16, and this was consistent with its greater anti-adhesive activity (Fig. 3A, B). This is unlikely to be a toxic effect as, to our knowledge, low molecular weight chitin based molecules do not have toxicity in animal systems.

### Conclusions and perspectives

Plants are able to distinguish between different LCO structures and oligo-chitins through specific interactions with different LysM receptors to impart developmental and innate immunity responses respectively [1], [7], [13], [54]. In addition, recent results show that chitin-recognising LysM receptors retain residual ability to interact with LCOs, but suppression of an innate immunity response resulted rather than induction [13]. The results presented here show that animal cells, like plants, can also distinguish between LCOs and oligo-chitins of comparable carbohydrate backbone size to impart different responses. This occurs even though chitin or chitin-like molecules are not known to be synthesised by mammals. Second, LCOs with different terminal residues can impart opposite biological effects upon mammalian ECs. Some impart anti-angiogenic effects and others pro-angiogenic effects. The differential activity of these LCOs is broadly consistent with the different structure-dependent responses imparted by LCOs and chitins in plants.

Higher concentrations of LCO and LCO-like compounds were required to trigger activity in mammalian cells than in plants. Despite this, the concentrations used still fall into a range that could be used in a therapeutic setting. For example, similar concentrations of the carbohydrate based therapeutic Muparfostat are used in successful clinical trials. In addition, our results also show there is a correlation between angiogenesis modulating activity in mammals and interactions of ECs with ECM components *in vitro*.

Unlike plants, a mammalian receptor for LCO, LCO-like and the shorter disaccharide molecules is not known. However, chitin-binding proteins and chitinases of the GH family, which are predicted to be secreted, are involved in several physiological processes in mammals [15], [16], [18], [55]. In addition, chitin oligomers have been shown to trigger intracellular kinase signalling cascades. Chitin induction of innate immune cells associated with allergic responses occurs independently of the Toll-like-receptor 4 (TLR4) and myeloid differentiation factor 88 (MyD88) [15]. Apart from the GH family of proteins, vertebrates also encode LysM domain containing proteins, several of which incorporate single pass transmembrane and GRAM (Glucosyltransferases, Rab-like GTPase activators and Myotubularins) domains [19], but not kinase domains. Chitin-binding proteins and chitinases of the GH family and mammalian LysM domain proteins should be explored as mammalian candidates for receptors for LCO and LCO-like molecules. Zebrafish might be a useful system to test LCO biological activity and tissue specificity in a vertebrate model. This is because (a) zebrafish produce endogenous chito-oligosaccharides that appear to play a role in early embryogenesis [14], [24] (although this is not demonstrated in mammals) and (b) zebra fish LysM domain and chitinase proteins show specific expression patterns in the nervous system during embryogenesis [19] and the accessibility of embryogenesis in this system has distinct advantages.

The pro-angiogenesis activities of compound 11, a de-acetylated chitin disaccharide, and compounds 7 and 9, are key findings since the efficacy of current pro-angiogenic therapies is not ideal [31], [32]. However, compound 11 lacks the lipophilic side chain found in LCOs and, although it may be resistant to chitinase degradation [52] the half-life of this compound *in vivo* is likely to be brief due to rapid excretion. Therefore, the routes for synthesising the novel lipophilic variants of compound 11 that we devised (see Methods S1 in File S1) are important, albeit this resulted only in anti-angiogenic derivatives being made. Of these, compound 14 displayed strong anti-angiogenic activity *in vitro* and further studies of structural analogues of this compound are warranted. Based on the disaccharide structures synthesised and their activity *in vitro*, a route to making chitin oligosaccharide compounds of potential therapeutic value has been identified and this should be of wide interest. Therefore, the derivatives of the disaccharide compounds identified in this study could be modelled and tested for therapeutic efficacy by using *in vivo* systems. Therefore, LCOs join a growing class of short carbohydrate-based molecules with therapeutic potential [56], [57].

## Supporting Information

S1 File **Supporting Information. Fig. S1**, Structure of artificial LCO-like compounds. **Fig. S2**, Details of the synthesis steps and reaction conditions used for the production of compounds 11, 13, 14 15 and 16. **Methods S1**, Experimental procedures for generating compounds 14, 15 and 16 and NMR spectra of all compounds used in synthesis reactions.
(DOCX)
