# Supplementary material for: Lipo-Chitin Oligosaccharides, Plant Symbiosis Signalling Molecules That Modulate Mammalian Angiogenesis In Vitro
Source: PLoS One. 2014 Dec 23;9(12):e112635. doi: 10.1371/journal.pone.0112635 (PMC4275186; doi:10.1371/journal.pone.0112635)
Supplement: S1 File — Supporting Information. Fig. S1, Structure of artificial LCO-like compounds. Fig. S2, Details of the synthesis steps and reaction conditions used for the production of compounds 11, 13, 14 15 and 16. Methods S1, Experimental procedures for generating compounds 14, 15 and 16 and NMR spectra of all compounds used in synthesis reactions. (DOCX) [file pone.0112635.s001.docx]

**Supporting Information**

**Djordjevic et al.**

**Lipo-Chitin Oligosaccharides, Plant Symbiosis Signalling Molecules That Modulate Mammalian Angiogenesis**

**Figure S1.** Structure of artificial LCO-like compounds. (A) The structure of the potent anti-angiogenic m-benzamide substituted compound 6. Compared to PI-88 (Muparfostat, a known anti-angiogenic compound; [1]) it significantly inhibited tube formation at 100, 10 and 1 μg/ml whereas PI-88 was active at 100 μg/ml only. (B). The structure of pro-angiogenic phenyl-acetyl substituted compound 7 (Table 1). (C). The structure of the inactive compound 10, which lacks reducing end fucosylation (Table 1).

**Figure S2**. Details of the synthesis steps and reaction conditions used for the production of compounds 11, 13, 14 15 and 16. a) 1. BnBr, NaH, DMF (78%) 2. TES, TFA, CH2Cl2, 0 °C (77%), b) **31** [2] AgOTf, NIS, CH2Cl2, -15 °C (78%), c) 1. aq. MeNH2 (40% wt), EtOH 2. Ac2O/pyr. (95%), d) AcSH, pyr. (91%), e) NaOMe/MeOH (**34**: 60%, **37**: 69%), f) aq. MeNH2 (40% wt), EtOH (53%), g) CAN, acetonitrile/water (**35**: 53%, **39**: 87%), h) Pd(OH)2/C, H2, THF/water (**13**: 31%, **11**: 9%), i) R1CO2H, PyBOP, Hünig’s base, DMF (**40**: 51%, **41**: 37%, **42**: 50%), j) Pd(OH)2/C, H2 , THF/water (**14**: 43%, **15**: 63%, **16**: 44%).

**Methods S1**

**General**: NMR spectra were recorded on a Bruker Avance III spectrometer at 500 MHz (^1^H) or 126 MHz (^13^C) and the spectra for all key compounds are listed below. Spectra were measured in either CDCl_3_, CD_3_OD or CDCl_3_/MeOD/D_2_O (7:4:0.6) using Me_4_Si as an internal reference. High resolution accurate mass determinations were performed on a Waters Q-Tof Premier mass spectrometer under electrospray ionisation conditions. Anhydrous solvents were obtained from Aldrich. Compound **29** was purchased from Carbosynth Ltd (8 & 9 Old Station Business Park, Compton, Berkshire, RG20 6NE, UK). Compounds **11** and **13** were synthesised as outlined below; analytical data of both compounds are in agreement with published data from an alternative synthetic approach [3].

**Experimental Procedures**:

*4-methoxyphenyl 2-azido-2-deoxy-2,6-di-O-benzylβ-D-glucoside* **30**

*4-*methoxyphenyl 2-azido-2-deoxy-4,6-benzylidene *β-D-glucoside* (**29**) (4.93 g, 12.3 mmol) and benzyl bromide (1.91 mL, 16 mmol) were dissolved in DMF (100 mL) and sodium hydride 60% (740 mg, 18.5 mmol) was added portionwise. The reaction was stirred at room temperature for 2h before the addition of methanol (50 mL). Solvents were removed under reduced pressure and the resulting solid was redissolved in dichloromethane. This organic phase was washed with ammonium chloride, water, brine, dried (MgSO_4_) and solvent removed. The crude product was recrystallised from ethyl acetate:petroleum ether to afford the intermediate benzyl ether as white needle-like crystals (4.73g, 78% yield). δ_H_ (CDCl_3_, 500 MHz) 7.47-7.49 (m, 2H), 7.38-7.39 (m, 5H), 7.32 (m, 3H), 7.02 (d, *J*=9.1 Hz, 2H), 6.84 (d, *J*=9.1 Hz, 2H), 5.60 (s, 1H), 4.93 (d, *J*=11.3 Hz, 1H), 4.82 (J=11.3 Hz, 1H), 4.81 (d, *J*=8.0 Hz, 1H), 4.37 (dd, *J*=4.6 and 9.9 Hz, 1H), 3.83 (t, *J*=10.3 Hz, 1H), 3.79 (t, *J*=9.3 Hz, 1H), 3.78 (s, 3H), 3.68-3.71 (m, 1H), 3.62 (t, *J*=9.2 Hz, 1H), 3.45-3.51 (m, 1H).

A solution of this benzyl ether (4.7g, 9.6 mmol) in dichloromethane (90 mL) was cooled to 0 °C under an argon atmosphere. To this was added triethylsilane (10.7 mL, 67.2 mmol), then trifluoroacetic acid (5.2 mL, 67.2 mmol) dropwise via syringe. The reaction was then stirred at 0 °C for 1h before dilution with dichloromethane. The organic phase was washed with saturated aqueous sodium bicarbonate (x3), water, brine, dried (MgSO_4_) and solvent removed under reduced pressure. Crude product was purified by column chromatography eluting with 20% ethyl acetate:petroleum ether to afford **30** as a yellow oil (3.64g, 77% yield). δ_H_ (CDCl_3_, 500 MHz) 7.28-7.41 (m, 10H), 7.04 (d, *J*=6.7 Hz, 2H), 6.80 (d, *J*=6.7 Hz, 2H), 4.94 (d, *J*=11.35 Hz, 1H), 4.79 (d, *J*=11.4 Hz, 1H), 4.74 (d, *J*=8.1 Hz, 1H), 4.57 (dd, *J*=11.9 and 20.4 Hz, 2H), 3.73-3.81 (m, 2H), 3.77 (s, 3H), 3.62-3.72 (m, 2H), 3.50-3.54 (m, 1H), 3.32 (t, *J*=8.9 Hz, 1H). δ_C_ (CDCl_3_, 126 MHz) 55.7, 65.6, 70.1, 71.8, 73.7, 74.3, 75.2, 82.5, 101.7, 114.6, 118.6, 127.7, 127.8, 128.1, 128.2, 128.5, 128.6, 137.8, 138.0, 151.2, 155.7.

*Synthesis of disaccharide* **32**

Thioglycoside **31** [2] (3.07 g, 6.25 mmol) and **30** (5.89 g, 12.65 mmol) were co-evaporated from dry acetonitrile and dried under vacuum for 1h. These were then dissolved in dichloromethane (100 mL) and cooled to -15 °C in a methanol/ice bath. *N*-Iodo-succinimide (2.39g, 10.62 mmol) was added and the reaction stirred for 5 min before the addition of silver trifluoromethanesulfonate (0.80 g, 3.12 mmol). The reaction was allowed to warm slowly and a rose colour developed at -10 °C. The reaction was stirred in the cool bath for 30 min and then at room temperature for a further 30 min. The reaction was diluted with dichloromethane and washed with saturated sodium bicarbonate: 10% sodium thiosulfate 1:1 v/v. The aqueous phase was extracted with dichloromethane and combined organics washed with brine, dried (MgSO_4_) and solvent removed under reduced pressure. The crude product was purified by column chromatography eluting with 30-50% ethyl acetate:petroleum ether to afford **32** as a pale yellow foam (4.40g, 78% yield). δ_H_(CDCl_3_, 500 MHz) 7.85 (d, *J*=5.4 Hz, 2H), 7.72 (d, *J*=5.4 Hz, 2H), 7.46 (d, *J*=7.1 Hz, 2H), 7.39 (t, *J*=7.6 Hz, 2H), 7.24-7.32 (m, 6H), 6.93 (d, *J*=9.1 Hz, 2H), 6.74 (d, *J*=9.1 Hz, 2H), 5.72 (t, *J*=9.1 Hz, 1H), 5.60 (d, *J*=8.4 Hz, 1H), 5.12 (t, *J*=9.2 Hz, 1H), 5.00 (d, *J*=11.4 Hz, 1H), 4.86 (d, *J*=11.4 Hz, 1H), 4.56 (d, *J*=8.2 Hz, 1H), 4.42 (d, *J*= 11.8 Hz, 1H), 4.37 (d, *J*=11.8 Hz, 1H), 4.28 (t, *J*=8.4 Hz, 1H), 4.08-4.14 (m, 2H), 3.85 (d, *J*=12.4 Hz, 1H), 4.08 (s, 3H), 3.62 (t, *J*=8.2 Hz, 1H), 3.54 (d, *J*=11.2 Hz, 1H), 3.43-3.46 (m, 3H), 3.38-3.41 (m, 1H). δ_C_ (CDCl_3_, 126 MHz) 20.4, 20.6, 20.7, 55.3, 55.6, 61.5, 65.7, 67.8, 68.6, 70.7, 71.8, 72.8, 74.6, 74.7, 75.0, 81.0, 97.2, 101.5, 114.5, 118.6, 123.6, 127.4, 127.5, 127.6, 128.3, 128.4, 131.4, 134.4, 138.1, 138.4, 151.1, 155.6, 169.4, 170.1, 170.6.

*Synthesis of disaccharide* **36**

A solution of **32** (700 mg, 0.77 mmol) in pyridine (10 ml) was treated with thioacetic acid (10 ml) and stirred at room temperature overnight. The reaction was diluted with dichloromethane, washed with saturated sodium bicarbonate solution, water, brine, dried (MgSO4) and solvent removed under reduced pressure. Purification by column chromatography eluting with 20-50% ethyl acetate:petroleum ether afforded **36** (649 mg, 0.7 mmol, 91% yield). δ_H_ (CDCl_3_, 500 MHz) 7.84 (d, *J*=3.9 Hz, 2H), 7.73 (d, *J*=3.9 Hz, 2H), 7.22-7.36 (m, 10H), 6.75 (d, *J*=8.2 Hz, 2H), 6.67 (d, *J*=8.2 Hz, 2H), 5.83 (t, *J*=9.8 Hz, 2H), 5.44 (d, *J*=8.4 Hz, 1H), 5.13 (t, *J*=9.4 Hz, 1H), 5.09 (d, *J*=5.9 Hz, 1H), 4.81 (d, *J*=11.8 Hz, 1H), 4.70 (d, *J*=11.8 Hz, 1H), 4.29-4.40 (m, 3H), 4.20-4.23 (m, 1H), 3.95-3.99 (m, 2H), 3.85-3.87 (m, 1H), 3.71 (s, 3H), 3.66-3.69 (m, 1H), 3.51-3.56 (m, 2H), 3.44-3.47 (m, 1H), 2.04 (s, 3H), 2.03 (s, 3H), 1.91 (s, 3H), 1.85 (s, 3H). δ_C_ (CDCl_3_, 126 MHz) 20.4, 20.6, 20.7, 23.4, 53.4, 55.1, 55.6, 61.6, 68.8, 70.4, 71.8, 72.9, 73.0, 74.3, 74.7, 96.9, 98.7, 114.4, 118.0, 123.7, 127.5, 128.2, 128.4, 131.3, 134.4, 138.2, 138.5, 151.1, 155.0, 169.5, 170.0, 170.1, 170.6.

*Synthesis of triol* **37**

A solution of **36** (640 mg, 0.69 mmol) in methanol (5mL) was treated with 30% wt sodium methoxide in methanol (15µL) and stirred at room temperature overnight. The solvent was removed under reduced pressure and the crude product purified by column chromatography eluting with 5%-20% methanol:chloroform to yield **37** (419 mg, 0.53 mmol, 76%). δ_H_ (CDCl_3_, 500 MHz) 7.78 (dd, *J*=3.0 and 5.4 Hz, 2H), 7.65 (dd, *J*=3.2 and 5.4 Hz, 2H), 7.21-7.31 (m, 8H), 7.17 (d, *J*=6.6 Hz, 2H), 6.74 (d, *J*=9.1 Hz, 2H), 6.64 (d, *J*=9.1 Hz, 2H), 6.03 (d, *J*=7.9 Hz, 1H0, 5.24 (d, *J*=8.3 Hz, 1H), 4.98 (d, *J*=5.8 Hz, 1H), 4.71 (dd, *J*=11.8 and 26.4 Hz, 2H), 4.39 (t, *J*=10.7 Hz, 1H), 4.32 (dd, *J*=11.8 and 27.7 Hz, 2H, 4.09 (dd, *J*=8.4 and 10.7 Hz, 1H), 4.04 (t, *J*=6.5 Hz, 1H), 3.89-3.93 (m, 2H), 3.70 (s, 3H), 3.61-3.66 (m, 2H), 3.50-3.53 (m, 2H), 3.43-3.46 (M, 1H), 3.23-3.25 (m, 1H), 1.90 (s, 3H). δ_C_ (CDCl_3_, 126 MHz) 23.3, 53.1, 55.6, 57.1, 61.6, 67.1, 69.0, 71.0, 71.8, 72.9, 73.1, 74.3, 74.7, 75.7, 97.2, 98.8, 114.4, 118.0, 123.5, 127.4, 127.5, 127.7, 127.9, 128.2, 128.4, 131.6, 134.2, 138.1, 138.2, 151.1, 155.0, 168.6, 170.7.

*Synthesis of amine* **38**

To a solution of **37** (415 mg, 0.52 mmol) in ethanol (15 mL) was added 40% wt solution of methylamine in water (15 mL) and the reaction was heated to reflux for 16h. The reaction was cooled and solvent removed under reduced pressure. The resulting oil was pre-adsorbed onto silica for purification by column chromatography eluting with 5-20% methanol:chloroform. The product isolated was shown by NMR analysis to have incomplete cleavage of the phthalimido group so was re-subjected to the reaction conditions above. Purification by column chromatography eluting with 5-10% methanol:chloroform yielded **38** (184 mg, 0.27 mmol, 53%). δ_H_ (MeOD, 500 MHz) 7.25-7.38 (m, 10H), 6.95 (d, *J*=9.0 Hz, 2H), 6.79 (d, *J*=9.0 Hz, 2H), 5.03 (d, *J*=8.2 Hz, 1H), 4.94 (d, *J*=11.0 Hz, 1H), 4.63-4.68 (m, 2H), 4.58 (d, *J*=11.7 Hz, 1H), 4.21 (t, *J*=9.2 Hz, 1H), 3.98 (t, *J*=8.4 Hz, 1H), 3.84-3.90 (m, 2H), 3.73-3.78 (m, 1H), 3.73 (s, 3H), 3.51 (dd, *J*=6.5 and 12.3 Hz, 1H), 3.41 (t, *J*=8.6 Hz, 1H), 3.25 (t, *J*=8.4 Hz, 1H), 3.20-3.22 (m, 1H), 2.81 (t, *J*=93.4 Hz, 1H), 1.93 (s, 3H). δ_C_ (MeOD, 126 MHz) 23.1, 56.1, 56.6, 58.9, 62.8, 69.6, 72.2, 74.3, 75.7, 75.9, 76.2, 76.5, 79.0, 100.7, 101.6, 115.6, 119.5, 128.7, 128.8, 128.9, 129.0, 129.4, 129.5, 129.6, 139.6, 152.9, 156.9, 173.5.

*Deprotection of anomeric position* **39**

To a solution of **38** (184 mg, 0.27mmol) in acetonitrile:water 5:1 (18 mL) was added ammonium cerium (IV) nitrate (302 mg, 0.55 mmol) and the reaction was stirred at room temperature for 4 days. As reaction was still not complete by TLC analysis a further one equivalent of ammonium cerium nitrate was added and stirring continued for a further 2h. The reaction mixture was poured directly onto a column for initial purification by silica chromatography eluting with 20% methanol:chloroform. Fraction containing the desired product were combined and re-columned on silica gel eluting with 10-15% methanol:chloroform to yield **39** (134 mg, 0.24 mmol, 87%). δ_H_ (MeOD, 500 MHz) 7.24-7.38 (m, 10H), 5.08 (d, *J*=3.3 Hz, 1H), 4.93 (d, *J*=10.7 Hz, 1H), 4.72 (d, *J*=8.2 Hz, 1H), 4.66 (d, *J*=11.9 Hz, 2H), 4.59 (d, *J*=11.8 Hz, 1H), 4.23 (t, *J*=9.8 Hz, 1H), 4.14-4.21 (m, 2H), 3.86-3.92 (m, 2H), 3.70-3.74 (m, 2H), 3.41-3.49 (m, 2H), 3.25 (t, *J*=9.7 Hz, 1H), 3.14-3.18 (m, 1H), 2.86 (t, *J*=8.2 Hz, 1H), 2.03 (s, 3H). δ_C_ (MeOD, 126 MHz) 22.8, 54.8, 58.6, 62.5, 69.9, 71.3, 72.2, 74.3, 74.5, 76.1, 76.7, 79.2, 80.1, 92.7, 99.2, 128.7, 128.8, 129.0, 129.4, 129.5, 129.6, 139.6, 173.8.

*Synthesis of disaccharide* **40**

To a solution of stearic acid (6 mg, 21.3 µmol) and **39** (8 mg, 14.2 µmol) in dimethylformamide (2 mL) was added diisopropylethylamine (12 µL, 71.1 µmol) and a precipitate formed. The pH of the reaction was checked to be above pH10 and then PyBOP (11 mg, 21.3 µmol) was added and stirring continued at room temperature under an argon atmosphere for 16h. Dichloromethane was added dropwise until solution became homogeneous and the reaction was stirred for a further 4h. After this time, solvent was removed under reduced pressure and the crude reaction mixture purified by column chromatography eluting first with 5% methanol:chloroform to remove main impurities and final traces of dimethylformamide, then with 3-20% methanol:chloroform to 20% methanol:ethyl acetate to yield **41** (6mg, 7.2 µmol, 51%). δ_H_ (MeOD, 500 MHz) 7.25-7.42 (m, 10H), 5.05 (d, *J*=10.7 Hz, 1H), 5.03 (d, *J*=3.7 Hz, 1H), 4.55-4.69 (m, 4H), 4.03-4.08 (m, 2H), 3.96-4.00 (m, 1H), 3.66-3.83 (m, 5H), 3.41-3.49 (m, 2H), 3.14-3.24 (m, 2H), 2.20-2.23 (m, 2H), 1.96 (s, 3H), 1.58-1.67 (m, 2H), 1.30 (b, 28H), 0.89 (t, *J*=6.9 Hz, 3H). δ_C_ (MeOD, 126 MHz) 14.4, 22.7, 23.7, 27.1, 30.4, 30.5, 30.6, 30.8, 33.1, 37.7, 54.7, 58.1, 63.1, 69.9, 71.9, 72.8, 74.3, 75.6, 76.6, 77.0, 78.7, 80.2, 92.6, 101.5, 128.7, 128.8, 129.0, 129.4, 129.5, 129.7, 139.9, 173.3, 176.7.

*Synthesis of final product* **14**

To a solution of **40** (12 mg, 14.5 µmol) in 1:1 tetrahydrofuran:water (2 mL) was added palladium hydroxide 20% on carbon (approx 2 mg) and the reaction was stirred under an atmosphere of hydrogen for 24h. After this time TLC analysis visualising with permanganate dip showed no starting material was present. The solvent was removed under reduced pressure and crude product purified by column chromatography eluting with 10% methanol:chloroform to yield **14** (4 mg, 43%). All spectra of this compound were run in 7:4:0.6 CDCl_3_:MeOD:D_2_O. δ_H_ (CDCl_3_/MeOD/D_2_O, 500 MHz) 5.12 (d, *J*=3.1 Hz, 0.7H α-anomer), 3.91 (dd, *J*=1.9 Hz, *J*=11.3 Hz, 1H), 3.79-3.88 (m, 3H), 3.76 (dd, *J*=8.4 Hz, *J*=10.1 Hz, 1H),3.57-3.74 (m, 4H), 3.36-3.48 (m, 3H), 2.19-2.30 (m, 2H), 2.01/2.02 (2s, 3H), 1.57-1.65 (m, 2H), 1.21-1.37 (bm, 28H), 0.89 (t, *J*=7.1 Hz, 3H). δ_C_ (CDCl_3_/MeOD/D_2_O, 126 MHz) 13.8, 22.1, 22.5, 25.6, 29.2, 29.3, 29.5, 29.6, 31.9, 36.4, 53.9, 55.5, 60.1, 62.4, 69.4, 69.9, 70.4, 72.2, 74.4, 74.8, 75.6, 76.4, 79.2, 79.9, 90.9, 95.9, 101.6, 172.6, 175.8. HRMS (ESI) calcd for C_32_H_60_N_2_O_11_ [M+Na]^+^ m/z 671.4095, found 671.4089.

*Synthesis of final product* **15**

To a solution of **39** (14 mg, 25 µmol) and m-tridecyloxybenzoic acid (12 mg, 37.3 µmol) in dimethylformamide (2 mL) was added diisopropylethylamine (22 µL, 124 µmol) and PyBOP (20 mg, 38 µmol). The reaction was stirred at room temperature under an atmosphere of argon for 16h. Solvent was removed under reduced pressure and purified by column chromatography eluting with 5% methanol:chloroform followed by 3% methanol:chloroform to yield  **41** (8mg, 9.2 µmol, 37%). To a solution of **41** (8 mg, 9.3 µmol) in 1:1 tetrahydrofuran:water (2 mL) was added palladium hydroxide 20% on carbon (approx 2 mg) and the reaction was stirred under an atmosphere of hydrogen for 24h. After this time TLC analysis visualising with permanganate dip showed no starting material was present. The solvent was removed under reduced pressure and crude product purified by column chromatography eluting with 5-20% methanol:chloroform to yield **15** (4 mg, 5.8 µmol, 63%). All spectra of this compound were run in 7:4:0.6 CDCl_3_:MeOD:D_2_O. δ_H_ (CDCl_3_/MeOD/D_2_O, 500 MHz) 7.42-7.46 (m, 2H), 7.34-7.38 (m, 1H), 7.05-7.08 (m, 1H), 5.09 (d, *J*=2.5 Hz, 0.7H α-anomer), 4.69 (d, *J*=8.5 Hz, 1H), 3.98-4.04 (m, 3H), 3.93 (dd, *J*=1.9, *J*=11.6 Hz, 1H), 3.81-3.88 (m, 2H), 3.69-3.80 (m, 4H), 3.54-3.57 (m, 2H), 3.41-3.48 (m, 2H), 2.00/2.01 (2s, 3H), 1.75-1.82 (m, 2H), 1.42-1.50 (m, 2H), 1.25-1.40 (bm, 18H), 0.89 (t, *J*=6.9 Hz, 3H). δ_H_ (CDCl_3_/MeOD/D_2_O, 126 MHz) 13.9, 22.1, 22.5, 25.9, 29.2, 29.5, 31.8, 36.4, 53.9, 56.2, 56.7, 60.2, 61.1, 68.4, 69.4, 69.9, 70.5, 72.4, 74.1, 74.7, 76.4, 79.1, 79.9, 90.8, 95.6, 101.6, 113.5, 118.1, 119.1, 129.6, 135.3, 159.3, 169.4, 172.7. HRMS (ESI) calculated for C_34_H_56_N_2_O_12_ [M+Na]^+^ m/z 707.3731, found 707.3722.

*Synthesis of final product* **16**

To a solution of **39** (20 mg, 35.5 µmol) and phenylacetic acid (11 µL, 88 µmol) in dimethylformamide (2 mL) was added diisopropylethylamine (31 µL, 178 µmol) and PyBOP (28 mg, 53 µmol). The reaction was stirred at room temperature under an argon atmosphere for 16h. The reaction was diluted with ethyl acetate and water was added. The layers were separated and the aqueous layer was evaporated under reduced pressure. Purification by column chromatography eluting with 5-15% methanol:chloroform, then 5% methanol:chloroform yielded **42** (12 mg, 18 µmol, 50%).

To a solution of **42** (12 mg, 18 µmol) in 1:1 tetrahydrofuran:water (2 mL) was added palladium hydroxide 20% on carbon (approx 2 mg) and the reaction was stirred under an atmosphere of hydrogen for 24h. After this time TLC analysis visualising with permanganate dip showed no starting material was present. The solvent was removed under reduced pressure and crude product purified by column chromatography eluting with 5-20% methanol:chloroform to yield **16** (3.5 mg, 7 µmol, 44%). All spectra of this compound were run in 7:4:0.6 CDCl_3_:MeOD:D_2_O. δ_H_ (CDCl_3_/MeOD/D_2_O, 500 MHz) 7.28-7.36 (m, 4H), 7.22-7.28 (m, 1H), 545.10 (d, *J*=3.1 Hz, 0.7H α-anomer), 3.89 (d, *J*=11.9 Hz, 1H), 3.84 (dd, *J*=3.3 Hz, *J*=10.5 Hz, 1H), 3.63-3.82 (m, 4H), 3.53-3.63 (m, 3H), 3.36-3.48 (m, 3H), 3.35-3.40 (m, 2H), 2.00/2.01 (2s, 3H). δ_C_ (CDCl_3_/MeOD/D_2_O, 126 MHz) 22.1, 43.0, 53.8, 55.8, 60.1, 61.1, 69.3, 69.9, 70.5, 72.2, 74.3, 74.8, 76.4, 79.2, 79.9, 90.9, 95.9, 101.2, 126.9, 128.5, 129.1,173.4. HRMS (ESI) calculated for C_22_H_32_N_2_O_11_ [M+Na]^+^ m/z 523.1904, found 523.1896.

Proton and NMR spectra and MS spectra for key compounds

The ^1^H and ^13^C spectra for synthesised compounds 11, 13-16 and their intermediates 29, 30, 32-39 follow in addition to the single mass spectral analysis of compounds 11, 13-16.

Literature cited

1. Parish CR, Freeman C, Brown KJ, Francis DJ, Cowden WB (1999) Identification of sulfated oligosaccharide-based inhibitors of tumor growth and metastasis using novel *in vitro* assays for angiogenesis and heparanase activity. Cancer Research 59: 3433-3441.

2. Kartha KPR, Field RA (1998) Iodine and its Interhalogen Compounds : Versatile Reagents in Carbohydrate Chemistry V. Synthesis of 1,2-trans-Linked 1-Thioglycosides From Per-O-Acetylated Glycoses. Journal of Carbohydrate Chemistry 17: 693-702.

3. Barroca-Aubry N, Pernet-Poil-Chevrier A, Domard A, Trombotto S (2010) Towards a modular synthesis of well-defined chitooligosaccharides: synthesis of the four chitodisaccharides. Carbohydrate Research 345: 1685-1697
